# Non-Lipid Effects of PCSK9 Monoclonal Antibodies on Vessel Wall

**DOI:** 10.3390/jcm11133625

**Published:** 2022-06-23

**Authors:** Sabina Ugovšek, Miran Šebeštjen

**Affiliations:** 1 Faculty of Medicine, University of Ljubljana, 1000 Ljubljana, Slovenia; sabina.ugovsek@student.uni-lj.si; 2 Department of Cardiology, University Medical Centre Ljubljana, 1000 Ljubljana, Slovenia; 3 Department of Vascular Diseases, University Medical Centre Ljubljana, 1000 Ljubljana, Slovenia

**Keywords:** PCSK9 monoclonal antibodies, inflammation, endothelial dysfunction, haemostasis, thrombosis, coagulation, fibrinolysis

## Abstract

Elevated low density lipoprotein (LDL) cholesterol and lipoprotein(a) (Lp(a)) levels have an important role in the development and progression of atherosclerosis, followed by cardiovascular events. Besides statins and other lipid-modifying drugs, PCSK9 monoclonal antibodies are known to reduce hyperlipidemia. PCSK9 monoclonal antibodies decrease LDL cholesterol levels through inducing the upregulation of the LDL receptors and moderately decrease Lp(a) levels. In addition, PCSK9 monoclonal antibodies have shown non-lipid effects. PCSK9 monoclonal antibodies reduce platelet aggregation and activation, and increase platelet responsiveness to acetylsalicylic acid. Evolocumab as well as alirocumab decrease an incidence of venous thromboembolism, which is associated with the decrease of Lp(a) values. Besides interweaving in haemostasis, PCSK9 monoclonal antibodies play an important role in reducing the inflammation and improving the endothelial function. The aim of this review is to present the mechanisms of PCSK9 monoclonal antibodies on the aforementioned risk factors.

## 1. Introduction

Cardiovascular events remain the leading cause of morbidity and mortality in the Western countries despite new therapeutic options [1]. The underlying pathology of cardiovascular disease is atherosclerosis [2]. There are abundant epidemiological, genetic and clinical studies proving a causal link between the development of atherosclerotic plaques and low density lipoprotein (LDL) cholesterol [3]. LDL carries 60–70% of serum cholesterol [4]. It transports cholesterol from the liver to the peripheral tissues [4]. The LDL particle contains an apolipoprotein B-100 (apoB-100) which enables selective binding of LDL to its receptor [4]. By binding to the LDL receptor in the liver, more than 70% of LDL is removed from the circulation [4]. Therapies that lower elevated lipid levels slow the progression of atherosclerosis and reduce cardiovascular events and death [5]. Statins and ezetimibe are the standard of care for the management of high LDL cholesterol levels [6]. Promising results have been shown with PCSK9 monoclonal antibodies, inclisiran, bempedoic acid, angiopoietin-like 3 protein (ANGPTL3) inhibitors, peroxisome proliferator-activated receptor (PPAR) β/δ agonists and liver X receptor (LXR) agonists [6]. Regardless of statins and new lipid-modifying drugs to lower LDL cholesterol, only 54% of patients have achieved their risk-based LDL cholesterol goal [7]. The main reasons for failure to achieve LDL cholesterol goals are poor treatment adherence and suboptimal use of more efficacious lipid-lowering regimens.

Another modifiable risk factor associated with cardiovascular events is lipoprotein(a) (Lp(a)), a plasma protein that consists of LDL cholesterol, apoB-100 and plasminogen-like apolipoprotein(a) (apo(a)) [8]. Lp(a) levels are genetically determined by the LPA gene and have high inter-individually variability, but intra-individually are stable throughout life [8]. Considering the European guidelines, patients with Lp(a) concentration ≥ 50 mg/dL are at high risk of developing cardiovascular disease [9].

LDL cholesterol as well as Lp(a) internalize and accumulate in the arterial wall [10]. LDL cholesterol enters the intima via the LDL receptor, whereas Lp(a) are dependent on Lp(a) plasma concentrations, Lp(a) particle size, blood pressure, and arterial wall permeability [10,11]. The first one is found mainly in atherosclerotic lesions and the second accumulates all over the intima, respectively [10]. Both are taken up by macrophages to produce foam cells and thus promoting the development of atherosclerotic plaques [10]. Nevertheless, Lp(a) carries more atherogenic risk than LDL cholesterol because the former also consists of all the atherogenic components of LDL cholesterol and apo(a). [12,13]. Lp(a), due to the homology with plasminogen, competes with it for the same binding sites on endothelial cells, which promotes intravascular thrombosis and inhibits fibrinolysis [14,15]. PCSK9 induces inflammation in atherosclerosis independently from its hyperlipidemic effect. In addition to ox-LDL accumulation, PCSK9 can directly induce the expression of inflammatory cytokines [16].

In the last few years there have been therapies that lower LDL cholesterol as well as Lp(a), namely PCSK9 monoclonal antibodies and inclisiran. In this review we are focusing on the former since PCSK9 monoclonal antibodies are known to reduce levels of LDL cholesterol and Lp(a), and influence cardiovascular morbidity and mortality as well [17,18]. On the other hand, studies with inclisiran are in progress [19].

Various studies indicate that lipid-lowering agents not only reduce lipid levels, but also have non-lipid effects. They are mainly involved in inflammation, endothelial function and haemostasis. The latter begins with platelet adhesion and aggregation, followed by the activation cascade of clotting factors. Therefore, the aim of the present review is to describe the influence of PCSK9 monoclonal antibodies on the aforementioned risk factors.

## 2. Inflammation

**Figure 1:**
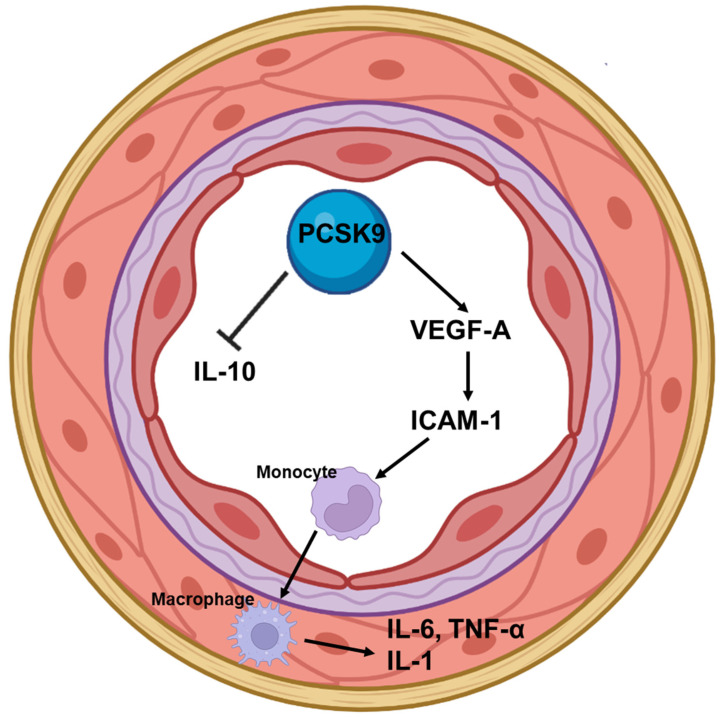
The role of PCSK9 in inflammation process. PCSK9 induces the expression of VEGF-A and ICAM-1 and, consequently, activates endothelial cells and stimulates monocyte/macrophage migration. The cascade promotes an inflammatory state and the progression of the atherosclerotic process. On the other hand, anti-inflammatory cytokines such as IL-10 attenuate the inflammatory response. PCSK9, proprotein convertase subtilisin/kexin type 9; VEGF-A, vascular endothelial growth factor A; ICAM-1, intracellular adhesion molecule-1; IL, interleukin; TNF-α, tumor necrosis factor-α.

Chronic inflammation plays an important role in the atherosclerotic process from endothelial dysfunction to plaque formation, its rupture and consequently arterial thrombosis, leading to acute cardiovascular events [20]. The most well studied biomarker for assessing inflammation and the most used in research and clinical practice is high sensitivity C-reactive protein (hsCRP) [21]. CRP is produced in the liver in response to proinflammatory cytokines such as interleukin (IL) 6, which is secreted by activated cells at the site of inflammation [22]. Other pro-inflammatory cytokines such as tumor necrosis factor-α (TNF-α), IL 8 and IL 18 aggravate inflammatory responses including the expression of adhesion molecules in endothelial cells, whereas anti-inflammatory cytokines such as IL 10 attenuate the inflammatory response [23]. In general populations without known cardiovascular disease, CRP was an independent predictor of cardiovascular events [24]. On the other hand, in patients with stable coronary artery disease with optimal medical therapy, inflammatory cytokine IL 6, but not hsCRP, was independently associated with future coronary events [25]. Statins are known to have anti-inflammatory effects and decreased mortality rates in patients with coronary artery disease. Justification for the Use of Statins in Primary Prevention (JUPITER) was the first trial which prospectively assessed the effects of statin versus placebo on rates of cardiovascular events [26]. In more than 17,000 apparently healthy men and women with elevated levels of hsCRP, rosuvastatin significantly reduced the incidence of major cardiovascular events, despite the fact that nearly all study participants had lipid levels at baseline that were below the threshold for treatment according to current prevention guidelines. Statin therapy is associated with a significant increase in plasma PCSK9 concentrations, irrespective of the type of statin, dose and treatment duration [27]. PCSK9 plays a crucial role in the indirect regulation of serum LDL cholesterol concentration by regulating the number of LDL receptors on hepatic cell surfaces [28]. The role of PCSK9 in the atherosclerotic process is not limited just to lipids homeostasis, but is also involved in the inflammatory cascade (Figure 1) [29].

Several epidemiological studies evaluated the association of PCSK9 with some inflammatory markers such as white blood cells and hsCRP [30,31]. In patients with stable coronary artery disease, PCSK9 was associated with monocyte subsets, especially with intermediate monocytes, which are characterized by CD14++CD16+ on their surface and express strong pro-inflammatory behaviors [5]. In patients treated with statins, these relationships were clear, while this was not the case in statin naïve patients, the second group being very small. In this study, no relationship was found between levels of PCSK9 and hsCRP. On the other hand, in patients with acute coronary syndrome, PCSK9 levels were associated with hsCRP [30]. They also found that PCSK9 levels did not predict future coronary events at one year, but it has to be pointed out that PCSK9 concentration increased over one year, and only 30% of patients were treated with statins at the time of the event. Contrary to their findings in association with the PCSK9 Serum Levels and Platelet Reactivity in Patients With Acute Coronary Syndrome Treated With Prasugrel or Ticagrelor (PCSK9-REACT) study [32], PCSK9 levels were found to predict future acute coronary events in patients with very similar baseline characteristics, including the proportion of patients treated with statins. We have to remember that hepatocytes are not the only source of PCSK9 and that PCSK9 is also produced in endothelial cells, monocytes and macrophages [29]. PSCK9 is not only produced locally, but it acts locally as it is linked to the chronic inflammatory state of the atherosclerotic plaque, what might be one of the factors involved in plaque progression and rupture [33]. PCSK9 monoclonal antibodies have no influence on hsCRP levels regardless of the PCSK9 inhibitor type, patient characteristics, concomitant treatment or treatment duration [34]. Similarly, in patients with elevated LDL and Lp(a) levels that were mostly already treated with statins, additional treatment with the PCSK9 inhibitor evolocumab did not alter either local inflammation in the arterial wall or systemic inflammation [35]. Contrary to this, in patients with coronary artery disease or familial hypercholesterolemia who do not take statins due to statin intolerance, treatment with alirocumab attenuates arterial wall inflammation without changing systemic hsCRP [36]. In both studies, local inflammation was measured using 18F-fluoro-deoxyglucose positron-emission tomography/computed tomography (18F-FDG PET/CT). Arterial 18F-FDG uptake correlates with arterial macrophage content [35]. The difference between these two studies was higher Lp(a) levels both at baseline and at the end of the study in the first study. Since Lp(a)-mediated cardiovascular risk is partly driven by pro-inflammatory oxidized phospholipids (OxPLs), which are abundant on the apo(a) tail of Lp(a) [12], we can assume that this difference may explain the persistent arterial wall inflammation. This is supported by ex vivo data that potent Lp(a)-lowering following AKCEA-APO(a)-LRx, but not modest Lp(a)-lowering combined with LDL cholesterol reduction following PCSK9 monoclonal antibodies treatment, reduced the pro-inflammatory state of circulating monocytes in patients with elevated Lp(a) [37]. The Global Assessment of Plaque Regression With a PCSK9 antibody in a Measured by Intravascular Ultrasound (GLAGOV) trial demonstrated that the addition of the evolocumab to patients with coronary artery disease already pretreated with statins had a favorable effect on progression of coronary atherosclerosis as measured by intravascular ultrasound (IVUS) [38]. The post hoc analysis evaluated the effect of evolocumab-treated patients according to the baseline hsCRP strata (i.e., patients were divided into three subgroups based on their hsCRP levels, <1, 1–3 and >3 mg/L) [39]. The ability of evolocumab to induce the regression of atherosclerotic plaque was not attenuated by the presence of enhanced systemic inflammation and was equal in all three hsCRP subgroups. The results showed that in patients treated with statins, which already have a positive effect on inflammatory parameters, regardless of residual inflammation, an additional reduction in LDL cholesterol, without affecting inflammatory parameters with evolocumab, had a positive effect on reducing atherosclerotic plaque. These results were further confirmed by the High-Resolution Assessment of Coronary Plaques in a Global Evolocumab Randomized Study (HUYGENS), which showed that evulocumab treatment increases the stability of the atherosclerotic bed by reducing the lipid core and increasing the fibrous cap thickness [40].

## 3. Endothelial Dysfunction

The endothelium is an active inner layer of the blood vessel and is indispensable for the regulation of vascular tone and the maintenance of vascular homeostasis [41]. Its functional impairment is characterized by an imbalance between vasodilators and contracting factors [41]. Endothelial dysfunction represents one of the first manifestations of atherosclerosis and is involved in plaque progression and atherosclerotic complications [41]. The most widely used non-invasive method for assessing endothelial function is with high-resolution external vascular ultrasound to measure flow-mediated dilatation (FMD) of the brachial artery during reactive hyperemia [41]. Given the fact that endothelial dysfunction represents a systemic disorder, the aforementioned technique correlates well with coronary FMD and strongly predicts future cardiovascular events [42]. The main reasons for endothelial dysfunction are exposure to oxidative stress and cardiovascular risk factors, including increased levels of cholesterol [43]. Several studies demonstrated an improvement in endothelial function after treatment with statins, independent of its lipid-lowering effects [41].

Besides statins, studies with PCSK9 monoclonal antibodies to evaluate the effects on endothelial function were performed. Maulucci et al. showed that in patients after myocardial infarction already treated with statins at high doses and ezetimibe, two-month therapy with evolocumab improves endothelial function proportional to LDL cholesterol reduction (r = 0.69; *p* = 0.006) [44]. There is no data regarding Lp(a) values in their patients since it was found that increased Lp(a) values are associated with decreased FMD [45].

Furthermore, Di Minno et al. observed an improvement in endothelial function after treatment with 140 mg of evolocumab every 14 days for 12 weeks in patients with familial hypercholesterolemia on top of maximally tolerated lipid lowering therapy [46]. FMD significantly increased at week 12 (10.63% ± 5.89) from baseline values (4.78% ± 2.27) (*p* < 0.001) [46]. At the same time, a parallel improvement in the reactive hyperemia index and reduction in LDL cholesterol levels was seen [46]. In fact, a decrease of LDL cholesterol was the only independent predictor for FMD improvement (ß = −0.846; *p* = 0.015). The decrease of Lp(a) in their study was 7%, which is statistically important (*p* = 0.002), but not predictive for FMD improvement. On the other hand, treatment with alirocumab for 10 weeks in the ALIROCKS trial showed a nominal amelioration (+41%), but no significant change of flow-dependent dilatation of the brachial artery [47]. The sample size and duration of treatment with PCSK9 inhibitors were similar among the mentioned studies. Differences in the results in the ALIROCKS trial compared with the previously mentioned two studies may be due to lower baseline values of LDL cholesterol, although limitations of the FMD method cannot be ignored. In Evaluation of Cardiovascular Outcomes After an Acute Coronary Syndrome During Treatment With Alirocumab (ODYSSEY Outcomes) [48], baseline Lp(a) and LDL cholesterol levels and their reductions by alirocumab predicted the risk of future coronary events in patients after recent in secondary prevention. In their study, the mean decrease of Lp(a) was 23%, the decrease being the greatest in upper quartiles.

A noninvasive magnetic resonance imaging methodology can also be used to assess endothelial cell function [49]. Leucker et al. measured coronary endothelial function with magnetic resonance imaging after six weeks of treatment with evolocumab in people living with human immunodeficiency virus infection and in patients with dyslipidemia with no human immunodeficiency virus infection [49]. There was a significant increase in coronary endothelial function in both groups of patients [49]. In addition, LDL cholesterol levels significantly decreased, but there was no significant change in inflammatory markers in either group [49].

The exact mechanism by which the PCSK9 inhibitor improves endothelial function remains unknown. It is possible that its effect is mostly mediated by the reduction of LDL cholesterol levels, nevertheless other mechanisms can play the same role [44]. Lipid-lowering treatments ameliorate oxidative stress and improve endothelial nitric oxide synthase [44]. In addition, the inhibition of PCSK9 induces the up regulation of the LDL receptors [50]. This might increase the LDL cholesterol binding affinity for the LDL receptor, leading to an improvement of endothelial function [51]. On the other hand, PCSK9 is associated with a macrophage-mediated inflammatory response and treatment with PCSK9 monoclonal antibodies leads to decreased monocyte migratory capacity and reduced inflammatory response [52,53].

Marques et al. investigated the effects of alirocumab 150 mg every 14 days in patients with familiar hypercholesterolemia on subendothelial infiltration of leukocytes, a critical step in the atherogenic process [54]. After an eight-week regimen, the suppressed leukocyte adhesion to the dysfunctional arterial endothelium was observed [54].

PCSK9 monoclonal antibodies attenuate the proinflammatory activation of endothelial cells and reduce the apoptosis of endothelial cells, smooth muscle cells and macrophages [55]. Besides that, PCSK9 monoclonal antibodies may have an effect on circulating endothelial progenitor cells (cEPCs) [56]. The latter are characterized by positivity for CD34, CD133 and vascular endothelial growth factor receptor-2 (VEGFR-2), and are involved in the vascular repair as a response to the endothelial injury [56,57]. Itzhaki et al. in their study with the ePCSK9 inhibitor showed a decline in LDL cholesterol levels and the activation of cEPCs, evident by the elevated expression of CD34+/VEGFR-2+ cells [56]. Treatment with PCSK9 inhibitor promotes cECPs activation and differentiation into endothelial cells, independent of LDL cholesterol regulation [56].

## 4. Haemostasis

### 4.1. PCSK9 and Platelets’ Function

It is well known hypercholesterolemia, in particular high native LDL cholesterol and oxidized LDL cholesterol (oxLDL) levels, is associated with an increased risk of atherosclerosis and thrombosis due to increased platelet biogenesis, turnover and activity [58]. Platelets interact with the thrombogenic subendothelial matrix of the ruptured atherosclerotic plaque and with subsequent activation and aggregation [32]. Platelet and endothelial cell activation results in increased P-Selectin expression (other name is CD62P), followed by increased levels of the soluble form of P-Selectin [59]. Besides P-Selectin, the soluble CD40 ligand and other platelet activation markers also play a role in inducing a procoagulant effect [60].

**Figure 2:**
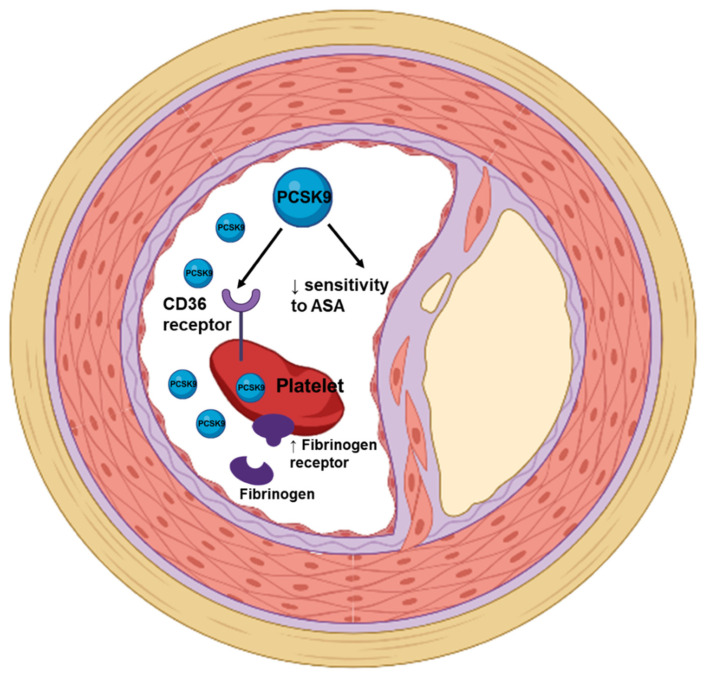
The role of PCSK9 in platelets’ activity. High PCSK9 levels enhance platelet activation and reduce platelet responsiveness to acetylsalicylic acid, thus promoting atherosclerotic events. ASA, acetylsalicylic acid.

**Figure 3:**
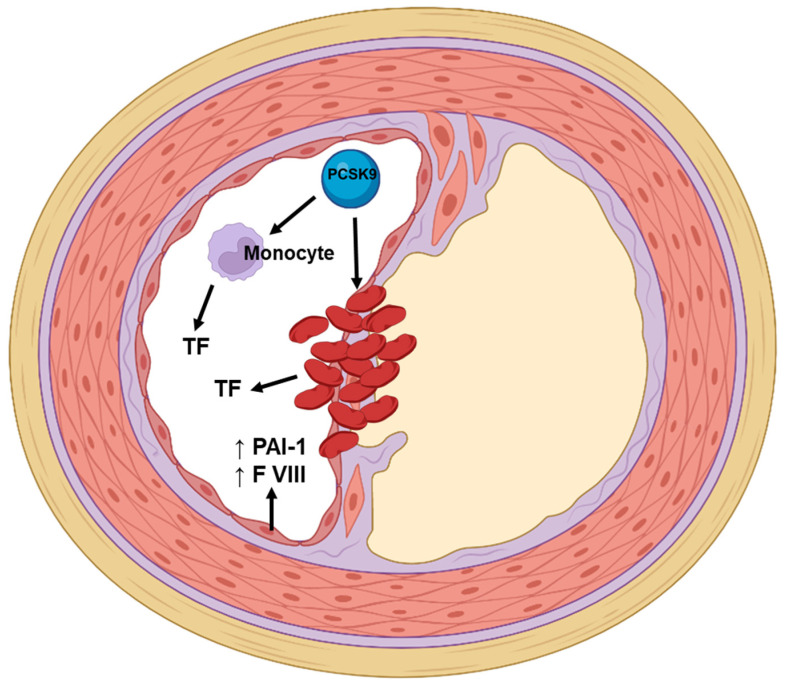
The role of PCSK9 in coagulation and fibrinolysis. PCSK9 induces the production of tissue factor, which is responsible for the activation of the extrinsic coagulation pathway and thrombus formation. TF, tissue factor; PAI-1, plasminogen activator inhibitor-1; F VIII, factor VIII.

Studies implicate not only increased LDL cholesterol but also that PCSK9 levels are involved in promoting platelet activation and coagulation (Figure 2 and Figure 3) independently of LDL cholesterol regulation [61,62,63].

In patients with stable coronary artery disease, a positive and independent relationship between plasma PCSK9 level and platelet count was observed [64]. Not only platelet count but also platelet activation was found to be associated with PCSK9 levels. In Pastori et al.’s study, the association between elevated PCSK9 and urinary 11-dehydro-thromboxane B_2_ (11-dh-TxB_2_), a stable metabolite of thromboxane A2 levels, suggested a role of PCSK9 in the regulation of platelet activation as well [65]. The potential mechanism underlying the connection between urinary 11-dh-TxB_2_ and PCSK9 might lead to the possible involvement of cyclooxygenase (COX)-1, an essential enzyme for thromboxane A2 [66]. In PCSK9-REACT study, patients with acute coronary syndrome after percutaneous coronary intervention were treated with prasugrel or ticagrelor [32]. Those with higher PCSK9 levels had increased platelet reactivity, they were low platelet responders with no difference between antiplatelet agents used and had a higher incidence of atherothrombotic events after one year [32]. Increased PCSK9 levels have a role as a predictor of higher platelet activation and cardiovascular events [66]. PCSK9 directly enhance platelet activation by binding to platelet CD36 and thus activating the downstream signaling pathways independently of its effect on lipids [67]. Both PCSK9 inhibitors and aspirin abolish the enhancing effects of PCSK9 on platelet activation. This data supports the use of aspirin in patients with increased PCSK9, and on the other hand confirms the antithrombotic effects of PCSK9 monoclonal antibodies.

Beside PCSK9 from plasma platelets, derived PCSK9 plays a significant role in atherothrombosis as a modulator of platelet activation [41]. Platelets store and release PCSK9 upon activation, which is enhanced in the presence of LDL cholesterol, not only ex vivo but also in patients with coronary artery disease. In the presence of PCSK9 antibodies platelet aggregation was significantly attenuated. As a result, in presence of PSCK9 antibodies, platelet-dependent thrombus formations significantly reduced [68]. Many studies indicate that statins [69] and PCSK9 monoclonal antibodies [70] have an effect on the hemostatic system. In approximately a third of patients, platelets show suboptimal response to antithrombotic therapy that can be linked to hypercholesterolemia [71]. It was shown that treatment with statins in patients with hypercholesterolemia significantly increased the aspirin mediated inhibition of platelet aggregation and thrombus formation, and this was beyond the lipid-lowering effect [71]. Barale et al. showed that in patients with primary hypercholesterolemia on a background of maximal tolerated statin and in the presence of concomitant therapy with acetylsalicylic acid, treatment with alirocumab or evolocumab significantly decreased platelet aggregation and activation [58]. In all hypercholesterolemia patients, decreased platelet membrane expression of CD62P and plasma levels of the in vivo platelet activation markers (soluble CD40 Ligand, Platelet Factor-4, and soluble P-Selectin) were observed [58]. Barale et al. in their study claimed that in patients with hypercholesterolemia the inhibition of PCSK9 with alirocumab or evolocumab results in increased platelet responsiveness to acetylsalicylic acid [58]. In the same study, patients with hypercholesterolemia treated with ASA were shown to have reduced platelet aggregation when stimulated with adenosine diphosphate (ADP), arachidonic acid and collagen compared to healthy subjects. Hence, we can infer that ASA reduces platelet aggregation in statin-treated patients with hypercholesterolemia. Patients with hypercholesterolemia who were also treated with statins but not ASA had increased platelet response to ADP compared to healthy subjects [58]. Treatment with PCSK9 monoclonal antibodies in patients not treated with ASA did not affect the response of platelets to the aforementioned substances. In contrast, treatment with PCSK9 monoclonal antibodies in patients previously treated with ASA resulted in decreased platelet responsiveness to all three substances. Platelet aggregation in high shear stress was measured with platelet function analyzer PFA-100. Closure time (CT) with collagen plus epinephrine did not differ between patients with hypercholesterolemia not treated with ASA and their healthy peers, while it was prolonged in hypercholesterolemic patients treated with ASA [58]. Treatment with PCSK9 monoclonal antibodies prolonged CT only in patients treated with ASA. Since both therapies, statins [72] and PCSK9 monoclonal antibodies, [58] improve the platelet response to ASA, it could be that a decrease in LDL cholesterol concentration directly affects platelet aggregation, but we need to consider the option that this effect goes beyond LDL cholesterol reduction. In addition to sensitizing platelet activation, dyslipidemia also seems to result in thrombocytosis, which ultimately elevates the risk for adverse thrombotic events [73]. However, the lowering of LDL cholesterol, regardless of how it is achieved, followed by a consequent reduction in thrombocytopenic counts, may lead to a reduced risk of thrombocytopenic events.

### 4.2. PCSK9 Monoclonal Antibodies and Coagulation and Fibrinolytic Parameters

Thrombosis which is unwanted extension of a haemostasis reaction occurs in both arterial and venous beds by different mechanisms. In arteries it is due to the atherosclerotic plaque rupture and consequential platelets activation, while in veins due to clotting activation [74]. PCSK9 monoclonal antibodies beside their main effect on LDL cholesterol decrease also moderately decrease Lp(a), which can be a common risk factor for arterial and venous thrombosis. Lp(a) has an effect on the coagulation pathway through the promotion of the expression of tissue factor (TF). TF initiates activation of the extrinsic coagulation pathway, which leads to thrombus formation [75]. Lp(a) is believed to promote atherothrombosis due to its homology with plasminogen. Due to this structural homology, Lp(a) can bind to plasminogen receptors on the surface of platelets and prevent the interaction between plasminogen and tissue plasminogen activator (tPA). Therefore, tPA cannot convert plasminogen to plasmin [76]. In a prespecified analysis of both ODYSSEY Outcomes [77] and Further Cardiovascular Outcomes Research with PCSK9 Inhibition in Subjects with Elevated Risk (FOURIER) [78] clinical trials, they sought to find an association between venous thromboembolism (VTE) and Lp(a) levels and the influence of PCSK9 monoclonal antibodies of future VTE. It was shown that patients with increased Lp(a) but not LDL cholesterol levels have an increased risk for VTE. In the ODYSSEY Outcomes randomized clinical trial, statin-treated patients with recent acute coronary syndrome received the PCSK9 inhibitor alirocumab [77]. In a prespecified analysis, a reduction of the risk of major peripheral artery disease events after treatment with alirocumab was observed (hazard ratio 0.69, 95% CI 0.54–0.89, *p* = 0.004) [77]. The effect was more evident among those with high levels of Lp(a) [77]. A similar but statistically nonsignificant relationship was observed between alirocumab and the occurrence of venous thromboembolism [77]. On the other hand, a post hoc analysis of the FOURIER trial treatment with PCSK9 inhibitor evolocumab demonstrated a 46% relative risk reduction in venous thromboembolism (hazard ratio 0.54, 95% CI 0.33–0.88, *p* = 0.014) [78]. Greater reductions in Lp(a) levels were associated with greater decreases in the risk of venous thromboembolism [78]. No relation between baseline LDL cholesterol levels and the magnitude of venous thromboembolism risk was observed [78]. In 685 consecutive patients with at least one episode of VTE and 266 sex- and age-matched healthy controls, serum levels of Lp(a) were found to be significantly higher in patients with previous VTE (49). No other established prothrombotic risk factors (activated protein C resistance, protein C, protein S, and antithrombin deficiency, and the factor V G1691A, MTHFR C677T, and prothrombin G20210A mutations) were found to be significantly combined with increased Lp(a). Elevated Lp(a) levels might contribute to the penetrance of thromboembolic disease in subjects being affected by other prothrombotic defects, such as FV G1691A mutation. Several case-control studies have shown increased VTE risk with elevated Lp(a) concentrations [79,80,81]. On the contrary, a population-based prospective study in 2180 middle-aged men without a history of VTE at the study entry showed no evidence of an association of circulating Lp(a) with the risk of VTE [82]. These results are similar to previous prospective studies in different populations [83,84,85,86]. No exact mechanism except lowering Lp(a) is known to be involved in the inhibition of coagulation or increasing of fibrinolysis by PCSK9 monoclonal antibodies. On the other hand, the potent reduction of Lp(a) with antisense oligonucleotides did not affect ex vivo fibrinolysis in humans [87], therefore, a decrease in Lp(a) is probably not the only factor influencing the reduction in VTE incidence. In order to determine the role of PCSK9 monoclonal antibodies in preventing VTE, we would need a study involving patients after VTE or at high risk of VTE. The research so far has included patients after a cardiovascular incident in the arterial system in which, although they share many common risk factors with patients with VTE, there are also significant differences.

In patients with familial hypercholesterolemia intolerant to statin treatment with either PCSK9 inhibitor did not decrease D-dimer or fibrinogen, which is one of the most robust clinical markers for decreased thrombogenicity [88]. An in vitro study showed that PCSK9 in a dose dependent manner induced TF production in peripheral blood mononuclear cells (PBMC), which can be inhibited with pretreatment with human anti-PCSK9 monoclonal antibody (mAb) [89]. Most importantly, the increase in TF procoagulant activity (TF PCA) is PCSK9 dose dependent without evidence of a plateau, and is also inhibited with PCSK9 mAB, while pretreatment with PCSK9 mAb has no effect on baseline TF PCA. The summary of the studies reviewed here is presented in Table 1.

## 5. Conclusions

A majority of the studies are focused on PCSK9 expressed in liver and its local function on LDLR. But we have to bear in mind that PCSK9 is also expressed in other cells and tissues [90]. It could be that the most important source of extrahepatic PCSK9 are cells in the arterial wall, in particular endothelial cells and smooth muscle cells, monocytes and macrophages. These cells are involved not only in the initiation and progression of atherosclerosis, but also in plaque rupture and consequent thrombus formation. Hence, not only local production, but mostly local utilization of PCSK9 is important. PCSK9 activates platelets, increases inflammation and prevails coagulation/fibrinolysis equilibrium to coagulation. On the other hand, Lp(a) possess the same properties in the atherosclerotic process. PCSK9 antibodies (alirocumab and evolocumab) that possess evidence on reducing cardiovascular morbidity and mortality decrease Lp(a) levels by the mechanisms not fully known and lower LDL cholesterol by increasing the number of LDLR. It was also found that both drugs decrease the incidence of VTE, which is associated with the decrease of Lp(a) values. One of the possible explanations would be that decreased levels of PCSK9 in circulation, and in particular in or near the atherosclerotic lesion or injured endothelium, are responsible for the lower incidence of acute arterial and VTE events. In the future it might be reasonable to measure the concentration of PCSK9 and not only levels of LDL cholesterol an Lp(a). Due to different mechanisms and the site of action of PCSK9 monoclonal antibodies and inclisiran in reducing PCSK9, the results of the ORION-4 study will be of particular interest [91]. ORION-4 is a double-blind randomized trial, which will answer the question of whether inclisiran reduces the risk of myocardial infarction and stroke in terms of safety and efficacy regarding hard clinical atherosclerotic cardiovascular disease endpoints. It would be most interesting to see if the results would be comparable to the results observed with both PCSK9 monoclonal antibodies. Since no direct comparison between inclisiran and PCSK9 monoclonal antibodies is to be expected, some answers could be obtained from the ORION-3 study. The ORION-3 study is an open-label, non-randomized, active comparator extension trial to assess the efficacy, safety, and tolerability of long-term dosing of inclisiran and evolocumab given as subcutaneous injections in participants with high cardiovascular risk and elevated LDL cholesterol (NCT03060577).

## Figures and Tables

**Table 1:** Overview of the studies that have evaluated the effects of PCSK9 monoclonal antibodies on inflammation, endothelial function and haemostasis. For full trial names and details, see main text.

Study	Study Population		Treatment	Primary Endpoint	Outcome
	(*n*)	Characteristics			
Cao et al. (2018) [34]	4198	FH or non-FH	A or E or LY3015014 or RG7652	Change in hsCRP	No beneficial changes in hsCRP
Stiekema et al. (2019) [35]	129	Elevated Lp(a)	E vs. placebo	Change in arterial wall inflammation	No beneficial changes in arterial wall inflammation, assessed as MDS TBR of the index vessel
Hoogeveen et al. (2019) [36]	50	Atherosclerotic disease or FH	A vs. placebo	Change in arterial wall inflammation	Reduced arterial wall inflammation assessed as MDS TBR of the index carotid (−6.1%)
Stiekema et al. (2020) [37]	18	Elevated Lp(a)	E	Change in gene expression and function of monocytes	No beneficial changes in pro-inflammatory state of monocytes
	14	CVD and elevated Lp(a)	AKCEA-APO(a)-L_RX_		Reduced pro-inflammatory state of monocytes (−17%)
GLAGOV [38]	968	Angiographic coronary disease	E vs. placebo	Change in percent atheroma volume	Reduced percent atheroma volume (−0.95%), total atheroma volume
HUYGENS [40]	161	Non-ST-segment elevation myocardial infarction	E vs. placebo	Changes in plaque composition	Increased fibrous cap thickness, decreased atheroma volume, lipid arc and macrophage index
Maulucci et al. (2018) [44]	14	Myocardial infarction	E	Changes in endothelial function	Increased FMD (+40%), brachial artery diameter and velocity time integral
Di Minno et al. (2020) [46]	25	FH	E	Changes in endothelial function, lipid profile and oxidation markers	Increased FMD and RHI, reduced 11-dehydro-thromboxane (−18%) and 8-iso-prostaglandin F2α (−17%)
ALIROCKS [47]	24	Indication for treatment with PCSK9 antibodies	A	Changes in endothelial function	No beneficial changes in FMD carotid intima-media thickness, fractional anisotropy of carotid artery, P-selectin and VEGF
Leucker et al. (2020) [49]	19	Patients with HIV	E	Changes in coronary endothelial function at rest and during isometric handgrip exercise	Increased coronary CSA and CBF, no beneficial changes in CRP, IL-6, INFγ, TNFα and CD163
	11	Dyslipidemia			
Marques et al. (2022) [54]	14	FH	A	Changes in inflammatory state, endothelial function and cardiovascular outcomes	Reduced activation of platelets and leukocytes, increased IL-10, reduced INFγ and soluble PCSK9
Itzhaki et al. (2020) [56]	26	CVD	A or E	Change in cEPC	Increased CD34+/CD133+ (+0.98%), VEGF receptor-2+ (0.66%) and PCSK9
Barale et al. (2019)[58]	24	Hyper- cholesterolemia	A or E	Change in platelet function	Reduced platelet aggregation and expression of CD62P, soluble CD40 ligand, platelet factor-4 and soluble P-selectin
Schwartz et al. (2020) [77]	18,924	ACS	A vs. placebo	Peripheral artery disease event or venous thromboembolism	Reduced risk of peripheral artery disease (hazard ratio 0.69), no beneficial changes in reducing the risk of venous thromboembolism
Marston (2020) [78]	27,564	Stable atherosclerosis, hyperlipidemia	E	Venous thromboembolism	Reduced risk of venous thromboembolism (hazard ratio 0.54)
Schol-Gelok et al. (2018) [88]	30	Statin-intolerant patients with FH	A or E	Change in D-dimer and fibrinogen	No beneficial changes in D-dimer and fibrinogen

A, alirocumab; ACS, acute coronary syndrome; apoB, apolipoprotein B; CBF, coronary blood flow; CSA, cross sectional area; CVD, cardiovascular disease; E, evolocumab; FH, familial hypercholesterolemia; FMD, flow mediated dilation; HIV, human immunodeficiency virus; hsCRP, high sensitivity C-reactive protein; IL, interleukin; INFγ, interferon γ; LDL-C, low density lipoprotein cholesterol; Lp(a), lipoprotein(a); MDS TBR, most diseased segment target to background ratio; PCSK9, proprotein convertase subtilisin kexin type 9; RHI, reactive hyperemia index; TC, total cholesterol, TNFα, tumor necrosis factor α;VEGF, vascular endothelial growth factor.

## Data Availability

Not applicable.
